# Non-Motor Symptoms after One Week of High Cadence Cycling in Parkinson’s Disease

**DOI:** 10.3390/ijerph16122104

**Published:** 2019-06-14

**Authors:** Sara A. Harper, Bryan T. Dowdell, Jin Hyun Kim, Brandon S. Pollock, Angela L. Ridgel

**Affiliations:** 1 Department of Medicine, Division of Gerontology, Geriatrics, and Palliative Care, University of Alabama at Birmingham, Birmingham, AL 35205, USA; 2 Center for Exercise Medicine, University of Alabama at Birmingham, Birmingham, AL 35205, USA; 3 Exercise Physiology Department, Kent State University, Kent, OH 44240, USA; bdowdell@kent.edu (B.T.D.); jkim74@kent.edu (J.H.K.); aridgel@kent.edu (A.L.R.); 4 Department of Exercise Science, Walsh University, North Canton, OH 44720, USA; bpollock@walsh.edu

**Keywords:** cognition, depression, exercise, neurodegenerative disease

## Abstract

The objective was to investigate if high cadence cycling altered non-motor cognition and depression symptoms in individuals with Parkinson’s disease (PD) and whether exercise responses were influenced by brain-derived neurotrophic factor (BDNF) Val66Met polymorphism. Individuals with idiopathic PD who were ≥50 years old and free of surgical procedures for PD were recruited. Participants were assigned to either a cycling (*n* = 20) or control (*n* = 15) group. The cycling group completed three sessions of high cadence cycling on a custom motorized stationary ergometer. The primary outcome was cognition (attention, executive function, and emotion recognition were assessed via WebNeuro^®^ and global cognition via Montreal Cognitive Assessment). Depression symptoms were assessed via Beck Depression Inventory-II. There was a main effect of time for emotional recognition (*p* = 0.048), but there were no other changes in cognition or depression symptoms. Regardless of intervention or Val66Met polymorphism, high cadence cycling does not alter cognition or depression symptoms after three sessions in one week.

## 1. Introduction

Parkinson’s disease (PD) is a progressive neurodegenerative disorder that is characterized by both motor and non-motor symptoms [1]. However, less may be understood regarding non-motor symptoms, such as cognition and depression symptoms [1,2,3]. There are also genetic variations as determinants of phenotype that may influence PD symptoms [4]. One gene that has been of recent interest in PD is brain-derived neurotrophic factor (BDNF)—a common single-nucleotide polymorphism where there is an amino-acid substitution in the prodomain of valine (Val) to methionine (Met) at codon 66 known as Val66Met polymorphism [5]. When present, it could lead to altered BDNF distribution [6] and decreased BDNF secretion [7]. Moreover, cascading effects could alter the BDNF regulation of synaptic transmission and neuronal growth [8] and the support of dopaminergic neurons in the substantia nigra [9]. Improvements in PD symptoms after exercise interventions have been associated with BDNF neuroplastic changes [10,11]. However, there is a high amount of inter-individual variability in the neuroplastic response to exercise [12] that may be influenced by BDNF Val66Met polymorphism.

With the prevalence of BDNF Val66Met polymorphism and non-motor symptoms, such as cognitive dysfunction (reduced attention and concentration, executive function, emotional recognition, and global cognition) and depression symptoms, in individuals with PD, there is a need for efficacious therapeutic modalities [1,2,3]. An aerobic exercise intervention, high cadence cycling, may be effective in improving non-motor symptoms without the established side effects that are associated with interventions such as PD prescribed medications, deep brain stimulation (DBS), and combined treatments [13,14].

Aerobic exercise can lead to increases in BDNF concentrations which could, in turn, decrease the prevalence of non-motor depression and cognitive dysfunction symptoms [15,16]. Although 86% of individuals with chronic diseases have observed increased peripheral BDNF concentration after exercise [17], individuals with Val66Met polymorphism may have diminished BDNF activity-dependent secretion [18,19].

Our group has shown that dynamic, high cadence cycling interventions are beneficial for individuals with PD motor symptoms [20,21]. This approach utilizes a motorized cycle to assist the rider in maintaining a high pedaling cadence, which improved motor symptoms after three exercise sessions, as described previously [20]. This intervention has a low to moderate intensity and has shown similar improvements to high-intensity interventions [20].

Given these improvements in PD motor symptoms, high cadence cycling could be a viable exercise intervention for improving cognitive domains and depression non-motor symptoms. Therefore, our central hypothesis was that three sessions of high cadence cycling would positively influence cognition and depression non-motor symptoms. A secondary hypothesis was whether cognition and depression non-motor symptoms would increase with the presence of Val66Met BDNF polymorphism.

## 2. Materials and Methods

### 2.1. Protocol

The cohort trial compared cognition and depression in individuals with PD between a high cadence cycling group and a no cycling control group. Prior to enrollment, all participants provided written informed consent approved by an Institutional Review Board. All research was conducted in accordance with the principles of the Belmont Report and was approved by the Kent State University Institutional Review Board (IRB # 15-605). The trial was registered with the Michael J. Fox Foundation at www.foxtrialfinder.org (trial 4345). Both groups were evaluated on Day 1 (pre-test) and returned 48 h after the third exercise visit for the cycling group (or Day 8 for the control) for post-testing.

### 2.2. Inclusion/Exclusion Criteria

Individuals were 50–85 years old, diagnosed with idiopathic PD, on prescribed PD-specific medications, and were free of contraindications to exercise. Contraindications included cardiovascular disease (heart attack, heart surgery, angioplasty, pacemaker, rhythm disturbance, heart valve disease, heart failure, heart transplantation, and congenital heart disease), stroke, and any surgical procedures for the treatment of PD (e.g., DBS). All individuals who met the inclusion criteria participated in a telephone pre-screening process using an American Heart Association (AHA)/American College of Sports Medicine (ACSM) exercise pre-participation questionnaire for Kent State University Exercise Physiology Laboratories [22]. Individuals that were identified as high risk were excluded from study participation. A family history of cardiovascular disease did not constitute a sufficient basis for exclusion. Following AHA/ACSM recommendations, individuals with two or more risk factors and/or were 80 years of age or older obtained the physician consent required by the Institutional Review Board.

### 2.3. Baseline Participant Characteristics

During this visit, participants’ height (DigiStad HM210D, Charder Medical, Tiachung City, Taiwan) and weight (Physician Balance Beam scale, Health o meter^®^ Professional, McCook, IL, USA) were measured. In addition, years of education, current PD prescription medications, and a baseline EQ-5D-3L quality of life questionnaire [23] were completed.

### 2.4. BDNF Val66Met Polymorphism

BDNF Val66Met polymorphism was tested for through a saliva test using an Oragene DNA collection kit (DNA Genotek^®^ Inc., Ottawa, ON, Canada) and outsourced to GenoFind (DNA Genotek Inc., Ottawa, ON, Canada) for analysis. The saliva samples had DNA extracted and then genotyped for a single-nucleotide polymorphism rs6262, or Val66Met. Quality checks were performed, including the PicoGreen analysis, Nanodrop absorbance readings, and agarose gel electrophoresis, for each sample. Previous investigations have utilized Genotek^®^ products to determine the BDNF allelic status for Val66Met polymorphism [24].

### 2.5. Intervention

The cycling group performed three, 40 min exercise visits as described previously [20]. Participants had a five-minute warm-up, a 30 min main-set, and a five-minute cool-down. Heart rate (HR), rating of perceived exertion (RPE) [25], power, and torque were recorded during the exercise intervention for the cycling group every second, separated into the warm-up, main-set, and cool-down blocks, and reported as the average ± SD [26]. The control group did not complete any cycling in the laboratory but were instructed to maintain normal levels of activity between the assessment visits.

### 2.6. Non-Motor Symptoms

#### 2.6.1. Cognition

Neurocognitive function was assessed through WebNeuro^®^ computer software (Brain Resource, Ultimo, New South Wales, Australia), which provides different clinical tests for the attention, executive function, and emotional recognition domains [27]. The attention and concentration domain involved a digit span and continuous performance test, while the executive function domain involved a maze task, switching of attention, verbal interference, and a go–no-go test as described previously [27,28]. Emotional recognition assesses variations in time and percent accuracy for identifying sad, disgust, fear, anger, happy, and neutral emotions [29,30]. When a participant was logged to be re-tested, an alternative test was presented. In addition, global cognitive function was evaluated via the Montreal Cognitive Assessment (MoCA, Greenfield Park, Quebec, Canada) [31,32]. To prevent a learning effect, alternative forms of MoCA were used in a counterbalanced manner. The MoCA is scored 0–30 where <26 may indicate a mild cognitive impairment [31,32].

#### 2.6.2. Beck Depression Inventory-II (BDI-II)

BDI-II [33] (The Psychological Corporation, San Antonio, TX, USA) was used to evaluate the prevalence and severity of depression symptoms [34,35] in individuals with PD [36]. Twenty-one questions were summed for a range of 0–63. The prevalence and severity of depression symptoms were classified with the following ranges: 0–13: minimal depression symptoms; 14–19: mild depression symptoms; 20–28: moderate depression symptoms; and 29–63: severe depression symptoms [33].

### 2.7. Statistical Analysis

All data were analyzed using the Statistical Package for Social Sciences software (IBM SPSS Statistics for Windows, Version 24.0, IBM Corp., Armonk, NY, USA). The alpha was set a priori to *p* ≤ 0.05. The baseline participant characteristics between groups were evaluated via independent samples t-tests. A repeated measures analysis of variance was performed to compare the cycling group’s physiological variables across the visits. The cognitive domains and depression symptom (non-motor) outcome measures were analyzed via two-way repeated measures analysis of variance comparing intervention groups over time. Val66Met BDNF polymorphism acted as a co-variate to potentially assist in predicting the physiological exercise outcomes and non-motor symptoms. The baseline characteristics are represented as the mean ± SD, *p*-value, and 95% CI. The cycling physiological variables are reported as the mean ± SD from each visit, *p*-value, and ηp^2^. The non-motor symptoms are reported as the *F*-value, *p*-value, and mean ± SD.

## 3. Results

Thirty-five participants completed the trial (cycling: *n* = 20 and control: *n* = 15). Two additional participants did not complete the cycling intervention and one participant returned for the post-testing after 8 days. Data from these participants were therefore removed from the final analysis. There was a statistically significant difference in the body mass index at the baseline likely driven by the variance in females by group (cycling: 9, 45% and control: 3, 20%). All remaining participant demographic characteristics were similar (Table 1). The exercise group recruitment was advertised primarily as an exercise research trial. Thus, recruitment may have engaged individuals participating in exercise and, therefore, those who had a lower body mass index.

Descriptive cycling physiological variables were not statistically significant between Visits 1 and 3 (Table 2). As noted in Table 2, there was variability as noted by the standard deviations across the outcomes across the three visits.

Mean results for Visits 1–3 were as follows: cadence 79.3 ± 5.6 rpm, power 1.92 ± 24.78, HR 86.25 ± 12.78 bpm, torque 2.21 ± 24.26, and RPE 11.0 ± 2.2. The HR and RPE values represent a low intensity exercise. There was a significant main effect of time for emotion recognition—*F* = 4.262, *p* = 0.048 (pre-control 0.04 ± 0.80 and cycling −0.36 ± 1.35, post-test control −0.28 ± 0.97 and cycling −0.48 ± 1.27). All other non-motor symptom outcomes were not significantly different—outlined in Table 3. There was no significant interaction for either between groups or over time.

## 4. Discussion

Although the concept that aerobic exercise can be beneficial for individuals with PD has been suggested, it was unknown if dynamic, high cadence cycling would alter non-motor symptoms. Therefore, our purpose was to investigate if high cadence cycling altered cognition and depression symptoms and whether potential changes are influenced by the presence of BDNF polymorphism. Our data indicate that there was a main effect of time for a subset of cognition—emotional recognition—regardless of intervention group or the presence of BDNF polymorphism. There were no significant differences in any of the other cognitive domains or depression symptoms. Thus, our overall results from this investigation do not support that novel high cadence cycling alters cognition or depression symptoms after three 30 min sessions.

### 4.1. BDNF Val66Met Polymorphism Role

There were reasons to suspect that the presence of Val66Met polymorphism would influence the non-motor symptoms. Met-allele carriers have been associated with a difficulty in the attention and concentration and executive function domains and global functioning compared to Val-allele carriers. Previous research suggests that the Val66Met presence was associated with more delayed recall errors compared to Val-allele carriers [37]. Moreover, another study found that the Val66Met group had higher verbal recall errors in the executive function domain [38]. In contrast, Foltynie and colleagues found that individuals with Val66Met had a better executive function performance than the Val-allele group [39]. Although our results observed no differences in attention and concentration, executive function, or global cognitive function, previous studies have found a strong correlation between Val66Met presence and mild cognitive impairment [40].

### 4.2. High Cadence Cycling Compared to High-Intensity Cycling

The current literature varies on whether acute changes in non-motor symptoms may occur with minimal exercise sessions. Participants performed at approximately 50–60% of their age-predicted maximal HR during the 30 min of high cadence cycling. In addition to the intensity, the duration of the intervention could further be reviewed. The ACSM states that adults should receive at least 150 min of moderate-intensity exercise per week [22]. In our investigation, participants exercised for 40 min for three exercise sessions totaling 120 min of aerobic exercise for one week. Further research is needed to investigation the ideal duration and intensity exercise to alleviate non-motor symptoms.

### 4.3. Possible Explanations

Previous research suggested that non-motor symptoms are prevalent among individuals with PD and that they may improve after aerobic exercise interventions, although this was not supported by our findings. Emerging literature has suggested that individuals with Parkinson’s disease may have a deficit in recognizing facial expression, or emotional recognition [41,42,43], hence why it was included in the non-motor assessment. Our past high cadence cycling research has primarily focused on motor symptom outcomes, such as rigidity and bradykinesia, in individuals with PD [20,21,44,45]. It is suggested that high cadence cycling may increase sensory feedback; activating basal ganglia circuits to enhance central motor processing may explain these favorable motor function results. However, it is possible that this approach is favorable for targeting motor symptoms, not non-motor symptoms [46,47,48]. Non-motor symptoms of PD are regulated by multiple non-dopaminergic neurotransmitters; thus, common levodopa and other pharmacological dopamine therapies may not address associated neurotransmitter dysfunction [48,49]. Interestingly, reports suggest that non-motor symptoms, such as depression, may have an “inconsistent relationship [with the] severity of motor symptoms” [50,51]. Thus, cell-based therapies that address the non-dopaminergic system may be better targeted approaches for non-motor symptoms in PD [48].

### 4.4. Study Limitations

Although this study yielded some interesting findings, recruiting individuals with PD and screening for various chronic health issues limits the implications for the PD population. All participants were tested while on their prescribed medication in order to not hinder their quality of life while participating in the investigation, and the timing of their medication was controlled as previously described [20]. Consequently, this approach means that our baseline data are a representation of the participants’ daily symptoms with PD medication. In addition, assessing BDNF blood concentration at both time points may have reflected whether BDNF was released during exercise [52]. Frazzita et al. observed that BDNF serum levels increased [53]. As alluded to in the discussion, participants cycled at a low intensity—even compared to past high cadence studies. Furthermore, many interventions are designed to achieve 150 minutes of aerobic exercise and are longer in length [10,54,55]. Briefly, cycling intervention lengths for other cycling paradigms tend to be three sessions a week for 8–12 weeks [10,54,55]. However, the paradigm proposed in this investigation has produced significant changes in motor symptoms after three sessions [20]. Previous research on improving depression symptoms in exercise interventions are varied. Research in older adults with Alzheimer’s disease suggests that participation in exercise plus behavioral intervention for 12 weeks can improve depression symptoms. Thus, a longer intervention may yield different non-motor symptom outcomes.

## 5. Conclusions

These results suggest that a short-term high cadence cycling intervention may improve emotional recognition over time but does not improve other cognitive domains or depression symptoms for individuals with PD. Furthermore, the Val66Met BDNF phenotype did not result in differential responses to this exercise intervention.

## 6. Patents

A.L.R. Inventor on US patent 9,802,081, 10,058,736 to Kent State University.

## Figures and Tables

**Table 1:** Baseline participant characteristics.

Variable	Cycling (*n* = 20)	Control (*n* = 15)	*p*-Value	95% CI
Age, years	65.05 ± 9.13	64.87 ± 6.90	*p* = 0.949	(−5.55, 5.92)
Gender, Female	9, 45%	3, 20%	*p* = 0.130	(−0.07, 0.58)
Val66Met Polymorphism	5, 25%	5, 33%	*p* = 0.602	(−0.402, 0.239)
BMI, kg/m^2^	26.15 ± 4.7	29.90 ± 4.3	*p* = 0.025 *	(−7.01, −0.51)
Education, years	15.3 ± 2.1	15.6 ± 2.0	*p* = 0.624	(−1.79, 1.09)
LED, mg	532 ± 275	560 ± 557	*p* = 0.847	(−268.48, 325.27)
EQ-5D QOL, points	6.9 ± 1.8	7.7 ± 1.8	*p* = 0.222	(−0.49, 2.02)
QOL VAS, %	72.58 ± 18.2	71.00 ± 14.0	*p* = 0.782	(−9.93, 13.08)

Independent samples *t*-tests compared cycling and control groups. Beck Depression Inventory-II (BDI-II) ranges from 0 to 63, ≥14 indicates mild or greater depression symptoms. Montreal Cognitive Assessment (MoCA) ranges from 0 to 30, 18–25 indicates mild cognitive impairment. Abbreviations: body mass index (BMI), Levodopa equivalent dose (LED), EQ-5D EuroQol Quality of Life (QOL), Quality of Life Visual Analog Scale (QOL VAS). Data indicate mean ± SD, *n*, or percentage. * *p* ≤ 0.05.

**Table 2:** Cycling physiological variables.

Variable	Visit 1	Visit 2	Visit 3	*p*-Value	ηp^2^
Cadence, rpm	80.3 ± 3.9	79.7 ± 4.4	78.0 ± 7.7	*p* = 0.811	0.019
Power	5.3 ± 23.6	0.7 ± 28.0	0.0 ± 23.7	*p* = 0.824	0.068
Torque, Nm	3.81 ± 20.19	−0.65 ± 25.13	3.34 ± 27.62	*p* = 0.630	0.057
Heart rate, bpm	84.5 ± 12.0	86.0 ± 13.0	88.4 ± 13.8	*p* = 0.584	0.042
RPE, Borg 6–20	11.0 ± 2.2	11.0 ± 2.6	11.2 ± 2.2	*p* = 0.566	0.100

Repeated measures analysis of variance compared cycling group visit physiological responses. Abbreviations: beats per minute (bpm), rating of perceived exertion (RPE), Newton meters (Nm), revolutions per minute (rpm). Data indicate mean ± SD.

**Table 3:** Outcome results.

Variable	Pre-Test	Post-Test	Statistical Results
Attention/Concentration	control 160.47 ± 30.36 cycling 167.60 ± 51.98	control 152.89 ± 47.47 cycling 164.92 ± 47.22	*F* = 0.164 *p* = 0.688
Executive Function	control 7612.75 ± 2232.06 cycling 7148.00 ± 2745.56	control 5912.83 ± 2999.25 cycling 6569.44 ± 2628.10	*F* = 0.400 *p* = 0.532
Emotional Recognition	control 0.04 ± 0.80 cycling −0.36 ± 1.35	control −0.28 ± 0.97 cycling −0.48 ± 1.27	*F* = 4.262 *p* = 0.048 *
MoCA	control 25.7 ± 3.2 cycling 25.7 ± 2.8	control 25.6 ± 3.3 cycling 25.0 ± 3.2	*F* = 0.614 *p* = 0.439
BDI-II	control 9.7 ± 7.5 cycling 9.45 ± 10.0	control 25.6 ± 3.3 cycling 25.00 ± 3.2	*F* = 0.837 *p* = 0.367

Repeated measures analysis of variance compared the cycling and control group non-motor symptoms over time. Attention/Concentration, Executive Function, and Emotional Recognition were assessed through WebNeuro^®^ software. Abbreviations: Beck Depression Inventory-II (BDI-II), Montreal Cognitive Assessment (MoCA). Data indicate mean ± SD. * *p* ≤ 0.05.
